# Potential Effect of Human Platelet Lysate on *in vitro* Expansion of Human Corneal Endothelial Cells Compared with Y-27632 ROCK Inhibitor

**DOI:** 10.18502/jovr.v16i3.9431

**Published:** 2021-07-29

**Authors:** Mohammad Amir Mishan, Sahar Balagholi, Tahereh Chamani, Sepehr Feizi, Zahra-Soheila Soheili, Mozhgan Rezaei Kanavi

**Affiliations:** ^1^Ocular Tissue Engineering Research Center, Research Institute for Ophthalmology and Vision Science, Shahid Beheshti University of Medical Sciences, Tehran, Iran; ^2^Blood Transfusion Research Center, High Institute for Research and Education in Transfusion Medicine, Tehran, Iran; ^3^Central Eye Bank of Iran, Tehran, Iran; ^4^Ophthalmic Research Center, Research Institute for Ophthalmology and Vision Science, Shahid Beheshti University of Medical Sciences, Tehran, Iran; ^5^National Institute of Genetic Engineering and Biotechnology, Tehran, Iran

**Keywords:** Cell Proliferation, Corneal Endothelial Cells, Human Platelet Lysate, ROCK Inhibitor

## Abstract

**Purpose:**

Corneal endothelial cell (CEC) therapy can be used as a promising therapeutic option for patients with various corneal endothelial dysfunctions. In this study, we compared the proliferative effect of human platelet lysate (HPL), as a xeno-free medium supplement, with Y-27632 Rho/rho-associated protein kinase (ROCK) inhibitor, as a well-known proliferative and adhesive agent for CECs, and fetal bovine serum (FBS) as the control, in the culture medium of human corneal endothelial cells (HCECs).

**Methods:**

We isolated HCECs from human donors and treated the cells as three different treatment groups including 20% HPL only, 10 μM Y-27632 ROCK inhibitor, combination of 20% HPL and 10 μM Y-27632 ROCK inhibitor, and 20% FBS as the control group. ELISA cell proliferation assay and cell counting was performed on the treated cells. Finally, HCECs were characterized by morphology and immunocytochemistry (ICC).

**Results:**

There was no significant proliferative effect of HPL on cell proliferation compared with the cells treated with Y-27632 ROCK inhibitor or the combination of HPL and Y-27632 ROCK inhibitor, but all the respected treatments had significant inducible effect on cell proliferation as compared with FBS-treated cells. The cells grown in all three treatment groups exhibited CEC morphology. Also, there was a higher expression of Na+/K+-ATPase and ZO-1, as CEC characteristic markers, in the culture of HCECs treated with HPL as compared with FBS.

**Conclusion:**

HPL offers a xeno - free and affordable medium supplement for CEC expansion that can be used in clinical applications.

##  INTRODUCTION

Corneal endothelium plays a pivotal role in the corneal transparency by regulating the flow of water from the aqueous humor into cornea.^[1]^ Corneal endothelial cell (CEC) density is above 2000 cells/mm ${}^{2}$ in healthy people; however, corneal endothelial dysfunction and abnormal corneal hydration can occur following a decrease in CEC density to fewer than 400 cells/mm ${}^{2}$ . ^[2]^ Loss of CECs also occurs as the result of Fuchs's endothelial corneal dystrophy (FECD) or following any form of corneal endothelial insult.^[1]^ Current treatments for these situations include penetrating keratoplasty (PKP) and endothelial keratoplasty techniques for replacing the damaged endothelial layer.^[3,4]^ These surgical procedures are invasive and have several side effects. More importantly, there is a need for an alternative solution based on cell therapy and/or tissue engineering to overcome the global shortage of donor corneas.^[5,6]^ Therefore, cell therapy based on cultivated human corneal endothelial cells (HCECs) from cadaveric donor corneas has been suggested in several studies.^[7,8]^

Rho GTPases family plays a pivotal role at many aspects of cell cycle progression.^[9]^ It was shown that inhibition of Rho/Rho-associated protein kinase (ROCK) signaling by selective Y-27632 ROCK inhibitor could promote the proliferation of several primary adult cells^[10,11,12]^ and also the CECs.^[13]^ Moreover, ROCK inhibitors have been introduced for several diseases such as pulmonary disease, cardiovascular disease, and cancer.^[14]^ Among several techniques introduced for expansion and adhesion of CECs for clinical purposes,^[15,16]^ the use of ROCK inhibitors has become popular.^[17,18]^ On the other hand, the platelet-rich plasma (PRP) as an autologous blood product contains several growth factors and signaling molecules needed for tissue regeneration, and it has attracted researchers' attention in this field.^[19,20,21]^ The use of platelet lysate obtained from PRP, as a xeno - free and accessible medium supplement, has also gained popularity and can be used based on Good Manufacturing Practice (GMP) standards.^[22,23,24]^

In this study, we aimed to investigate the effect of human platelet lysate (HPL) as a valuable source of growth factors on the proliferation behavior of HCECs as compared with Y-27632 ROCK inhibitor.

##  METHODS

To conduct the study, full ethical approval was obtained from the Institutional Review Board of the Central Eye Bank of Iran and the Ethics Committee of the Ophthalmic Research Center, Research Institute for Ophthalmology and Vision Science, Shahid Beheshti University of Medical Sciences, Tehran, Iran.

### Tissue Preparation

Twelve human pre-stripped Descemet's membrane (DM) with CEC density of > 2500 cells/mm ${}^{2}$ and preserved in Optisol-GS (Chiron Vision, Irvine, CA, USA) at 4°C were provided from the Central Eye Bank of Iran (Tehran, Iran). The corresponding donors were aged 26–50 years and the death to preservation time was < 30 hr. The preparation of pre-stripped DM endothelial keratoplasty tissue at the Central Eye Bank of Iran has previously been described.^[25]^ Briefly, after making a circumferential incision to the trabecular meshwork, without the use of dye or trephine, the edge of the DM from one side was gently grasped and peeled toward the opposite side and then transferred to Optisol-GS at 4°C.

### Human Platelet Lysate (HPL) Preparation

PRPs were provided from Iranian Blood Transfusion Organization (Tehran, Iran). The samples were pooled from three different healthy donors with a platelet count of 2.4 × 10 ${}^{5}$ /μl. HPL was obtained according to the repeated freeze-thaw method.^[26]^ Accordingly, the pooled PRPs were kept at - 80°C for 24 hr, then thawed at 37°C in a water bath for 1 hr, and this action was repeated three times. After that, the solution was centrifuged at 2000 g for 10 min to remove the debris. Finally, the supernatant containing HPL was filtered using a 0.2 μm sterile filter.

### Isolation and Culture of HCECs

Our experiment on cultivated HCECs was composed of two main parts (ELISA in part one and cell counting, morphology, and immunocytochemistry [ICC] in the second part). Each part was performed in triplicate. For each run, the DMs from six different cornea donors were pooled and incubated in 3.5 mg/ml collagenase A (Roche, USA) at 37°C for 50 min and then centrifuged at 300 g for 5 min. Finally, the pooled cells were cultured in DMEM: F12 supplemented with 20% fetal bovine serum (FBS; Gibco), 120 mg/ml penicillin (Sigma, Germany), and 220 mg/ml streptomycin (Sigma, Germany) on a 24-well plate coated with 20 mg/ml of fibronectin (Sigma, USA) at 37°C and 5% CO ${}_{2}$ . All experiments were performed when the cells were in P1. For this purpose, after two weeks of cell isolation and culture, the cells were passaged by trypsin/EDTA and seeded on a 24-well plate at a seeding density of 1 × 10 ${}^{4}$ cells per well with DMEM: F12 culture medium supplemented with three different treatment groups including 20% HPL, 10 μM Y-27632 ROCK inhibitor (STEMCELL Technologies, USA), combination of 20% HPL and 10 μM Y-27632 ROCK inhibitor, and 20% FBS as the control group. The concentration of Y-27632 ROCK inhibitor was designated based on the previous studies.^[18,27,28]^

### ELISA Cell Proliferation Assay

HCECs were seeded on 96-well plates at 5000 cells/well in 200 μl culture medium. The culture medium in each well was first changed with 100 ml of DMEM: F12 1:1 and then supplemented with 20% HPL, 10 μM Y-27632 ROCK inhibitor, combination of 20% of HPL and 10 μM Y-27632 ROCK inhibitor, and 20% FBS (control group). To determine whether the HPL and/or Y-27632 ROCK inhibitor altered cell proliferation, bromodeoxyuridine (BrdU) was added after 24 hr of treatment and the proliferation assay was performed according to the manufacturer's instructions (Roche Diagnostic, Mannheim, Germany). An ELISA reader (ELx 808 Absorbance Reader, BioTek Instruments, Winooski, VT) was implemented to read the absorption of the investigated samples at specified wavelengths.

### Cell Counting

To assess HCECs proliferation after treatments, phase contrast micrographs at × 100 magnification of three random photos were recorded and the number of cells were counted using ImageJ software (National Institutes of Health) and then averaged.^[29]^

### HCECs Characterization

The characteristics of the cultured HCECs were verified based on the morphology and the expression of molecular markers. Polygonal/hexagonal appearance as the characteristic of CECs, was used to differentiate these cells from human corneal stromal fibroblasts with spindle-shaped appearance (Olympus IX71, Tokyo, Japan). Also, the expression of the molecular markers was detected using ICC.

### Immunocytochemistry (ICC)

Two groups of treatments, the cultured HCECs with 20% HPL and 20% FBS, in a 24-well plate were fixed by incubating in –10°C methanol for 10 min. Then, the cells were permeabilized using Triton X-100 (0.25%) and blocked in 1% bovine serum albumin (BSA) in PBS for 1 hr at room temperature. The expressions of Na ${}^{+}$ /K ${}^{+}$ -ATPase, zonula occludens-1 (ZO-1) and vimentin were detected at protein levels by 1:500 of primary Rabbit anti-Human Na ${}^{+}$ /K ${}^{+}$ -ATPase (Santa Cruz Biotechnology Inc., Dallas, USA, sc-28800) and 1:400 of primary Rabbit anti-Human ZO-1 (Santa Cruz Biotechnology Inc., Dallas, USA, sc-10804) for 90 min each, and 1:800 of primary Rabbit anti-Human vimentin (Santa Cruz Biotechnology Inc., Dallas, USA, sc-5565) for 60 min. Thereafter, the slides were incubated with 1:200 of goat anti-rabbit IgG-fluorescein isothiocyanate (FITC) conjugated antibody (Santa Cruz Biotechnology Inc., Dallas, USA, sc-2012) for 45 min in darkness and at room temperature. After washing with PBS, the slides were counterstained with 1.5 mg/ml of 4,6-diamidino-2-phenyindole dihydrochloride (DAPI, Santa Cruz, USA) for 10 min. The images were then captured using a fluorescence microscope (Olympus IX71, Tokyo, Japan) equipped with a digital camera (Olympus U-TV0.63XC; Tokyo, Japan) and an excitation wavelength of 450–520 nm. ImageJ software (ImageJ 1.48; National Institute of Health; http://rsb.info.nih.gov/ij/) was used to quantify the corrected total cellular fluorescence (CTCF) per image and the mean values were compared between the HPL-treated and FBS, as the control cells.

### Statistical Analysis

Data were quantitatively compared between the treated groups using one-way ANOVA and Tukey's multiple comparison test for ELISA cell proliferation assay and cell counting, and t-test for CTCF analysis by the Graph Pad Prism (version 6.0). Results were expressed as the means ± standard deviation (SD) obtained from three independent experiments.

##  RESULTS

### Cell Proliferation Assay

**Figure 1:**
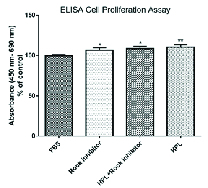
Cell proliferation assay. Results show a higher proliferative effect of all treatment groups as compared to the control group (*P < 0.05 and **P < 0.01).

HPL at 20% concentration significantly increased the cell proliferation rate compared with the control cultures in 20% FBS-containing medium. However, the proliferation of cultured HCECs after 24 hr treatment with HPL was similar to those treated with Y-27632 ROCK inhibitor and also with combination of HPL and Y-27632 ROCK inhibitor. Besides, Y-27632 ROCK in a concentration of 10 μM significantly increased HCECs as compared to 20% FBS control [Figure 1].

**Figure 2:**
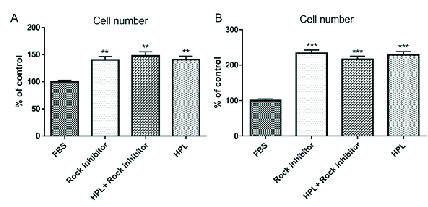
The numbers of HCECs after (A) one and (B) five days of treatments. As illustrated, the numbers of HCECs treated with HPL, combination of HPL and Y-27632 ROCK inhibitor, and Y-27632 ROCK inhibitor alone was significantly higher than FBS (control)-treated group (**P < 0.01 and ***P < 0.001).

**Figure 3:**
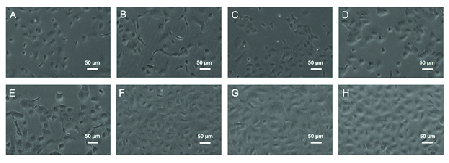
Representative photomicrographs of cultivated HCECs in treated and control groups (A–D) one and (E–H) five days after treatments. The morphology of HCECs after five days of cultures in (E) 20% FBS, (F) 10 μM Y-27632 ROCK inhibitor, (G) combination of 20% HPL and 10 μM Y-27632 ROCK inhibitor, and (H) 20% HPL.

**Figure 4:**
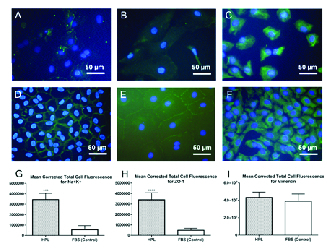
Expression of Na+/K+-ATPase, ZO-1 and vimentin proteins in the HCECs cultured in 20% HPL and 20% FBS, as the control group, after five days of treatments. (A) A merged FITC-Na+/K-ATPase antibody and DAPI image in the cells treated with 20% FBS. (B) A merged FITC-ZO-1 antibody and DAPI image in the cells treated with 20% FBS. (C) A merged FITC- vimentin antibody and DAPI image in the cells treated with 20% FBS. (D) A merged FITC-Na+/K-ATPase antibody and DAPI image in the cells treated with 20% HPL. (E) A merged FITC-ZO-1 antibody and DAPI image in the cells treated with 20% HPL. (F) A merged FITC-vimentin antibody and DAPI image in the cells treated with 20% HPL. Mean CTCF values of (G) Na+/K+-ATPase and (H) ZO-1 protein expressions show a significant increase in the HPL-treated HCECs as compared to the control group (***P < 0.001 and ****P < 0.0001, respectively). (I) Means of CTCF for vimentin expression were not significantly different between the two groups.

### Cell Counting

After one and five days, the number of cells was recorded from respected photos using ImageJ software and results showed that the cell numbers were significantly higher in all three treatment groups compared with FBS as the control group, at both time points as shown in Figure 2.

### Morphology 

The morphologies of cultured HCECs in DMEM/F12 medium supplemented with all three treatment groups and FBS as the control are shown in Figure 3. Polygonal/hexagonal shape, characteristics of CECs, were observed in the cultures, as it was confirmed with immunostaining for Na+/K+-ATPase [Figure 4D] and ZO-1 [Figure 4E] markers. The cells reached over 80% confluency after five days of culture.

### Immunocytochemistry (ICC)

The ICC results revealed that the cultivated cells were immune reactive for Na+/K+-ATPase [Figure 4D] and ZO-1 [Figure 4E] markers, confirming the identity of the cultured cells as CECs. Na+/K+-ATPase expression was detected in approximately 100% of the HCECs, and > 94% and > 98% of the HCECs expressed ZO-1 and vimentin, respectively. CTCF results showed higher expressions of the HCECs for Na+/K+-ATPase [Figure 4G] and ZO-1 [Figure 4H] proteins in 20% HPL-treated HCECs as compared to the FBS- treated (control) group. Also, means of CTCF for vimentin expression were not significantly different between the two groups [Figure 4I]. Taken together, these results indicate that the cells expressed the original characteristics of CECs. The negative controls without primary antibody did not show any FITC reactivity (data were not shown).

##  DISCUSSION

The results of this study indicate that HCECs gained a potential proliferation after treatment with 20% HPL in comparison with FBS as the control group although this proliferation effect was comparable to that in the Y-27632 ROCK inhibitor-treated group. To the best of our knowledge, there is no published investigation comparing the effects of HPL with Y-27632 ROCK inhibitor on HCECs culture. Given that HPL can be of GMP grade and is a cost-benefit supplement for cell culture, it can be a superior proliferative agent for *in vitro* expansion of HCECs for therapeutic goals.

More importantly, HPL was provided using a freeze/thaw method in the current study. It was previously demonstrated that this method ensures maximum release of growth factors and cytokines from the platelet compartment, providing enrichment of growth factors such as platelet-derived growth factor (PDGF)-AB/BB, platelet factor-4, epidermal growth factor (EGF), transforming growth factor (TGF)-β1, and fibroblast growth factor (FGF)-2.^[30]^

The proliferative potential of HPL, as an alternative to FBS, has been demonstrated for various primary cells to avoid risk of zoonosis for cell banking and clinical purposes.^[31,32]^ In addition, it was demonstrated that bovine CECs expanded in platelet releasate had good intercellular adhesion together with hexagonal morphologies.^[33]^ In another study, it was observed that all three different HPLs prepared via different ways induced proliferation of the BCE C/D-1b cells as a bovine CEC line, without noticeable differences compared to 10% FBS.^[34]^ Therefore, HPL has been introduced as a xeno - free supplement for culturing CECs^[16]^ and according to the results of our study, HPL induces a higher proliferative effect on the expansion of HCECs than FBS.

ROCK signaling pathway is involved in the regulation of important cellular functions including cytoskeleton organization, cell proliferation and migration,^[35,36]^ and initiation of apoptotic signaling pathways in stressful conditions.^[37,38]^ Also, this signaling pathway negatively regulates cell adhesion through actin depolymerization inhibition; however, it was demonstrated that inhibition of ROCK by Y-27632 agent promotes actin reorganization and results in CEC adhesion induction.^[8,13]^ More studies have suggested that Y-27632 ROCK inhibitor is a safe and effective additive agent for expansion of CECs.^[7,13,28,39]^ Interestingly, it was observed that Y-27632 improved the attachment and proliferation of HCECs isolated from young donors; however, these effects were not observed in HCECs isolated from older donors aged 60 and above.^[18]^ This may be due to the increase of cyclin kinase inhibitors p16  INK 4a and p21  WAF 1/ Cip 1 that results in an age-dependent increase in negative regulation of cell cycle.^[40]^ In the current study, although Y-27632 ROCK inhibitor exerted an elevated proliferation of HCECs in culture as compared with the control group, its effect was not superior to that of 20% HPL. Surprisingly, the combination of HPL and Y-27632 ROCK inhibitor had a similar effect on cell proliferation compared with Y-27632 ROCK or HPL alone. Further investigation will be required to verify the proposed statement.

In addition to the superior effects of the HPL over FBS on the cell proliferation, this natural PRP-derived supplement has also other advantages. It can be prepared simply as an autologous blood product from the recipients of HCECs allografts, which may be of great importance in reducing the risk of rejection and the need for immune suppression strategies.^[41,42]^ Moreover, given that PRP is an ideal source of autologous growth factors,^[41]^ it can be a proper agent for corneal endothelial tissue engineering and cell-based therapies in near future.

In conclusion, HCECs culture data showed a higher proliferation rate in the cells treated with 20% HPL in comparison to those treated with FBS. On the other hand, the effects of HPL on the proliferation of the HCECs were comparable with Y-27632 ROCK inhibitor alone and with the combination of HPL and Y-27632 ROCK inhibitor. Therefore, HPL can be used as a cost-benefit and xeno-free supplement in the culture of HCECs rather than Y-27632 ROCK inhibitor or FBS. Other advantages of HPL are its autologous and GMP grade characteristics that can ensure its safety in future clinical cell-based therapies. However, further investigations based on animal models and clinical trials are needed to elucidate the efficacy of HPL in comparison with Y-27632 ROCK inhibitor *in vivo*.

##  Financial Support and Sponsorship

The manuscript was funded by Shahid Beheshti University of Medical Sciences (Grant Number: 15739-5).

##  Conflicts of Interest

All authors declare that they have no conflicts of interests.
